# Deep Brain Stimulation in Anorexia Nervosa: Hope for the Hopeless or Exploitation of the Vulnerable? The Oxford Neuroethics Gold Standard Framework

**DOI:** 10.3389/fpsyt.2017.00044

**Published:** 2017-03-20

**Authors:** Rebecca J. Park, Ilina Singh, Alexandra C. Pike, Jacinta O. A. Tan

**Affiliations:** ^1^OxBREaD Research Group, Department of Psychiatry, University of Oxford, Oxford, UK; ^2^Neuroscience Ethics and Society Research Group, Department of Psychiatry, University of Oxford, Oxford, UK; ^3^Oxford Uehiro Centre for Practical Ethics, University of Oxford, Oxford, UK; ^4^Swansea University Medical School, Swansea University, Swansea, UK

**Keywords:** anorexia nervosa, deep brain stimulation, capacity, patient advocacy, neuroethics, clinical trial, neuromodulation

## Abstract

Neurosurgical interventions for psychiatric disorders have a long and troubled history (1, 2) but have become much more refined in the last few decades due to the rapid development of neuroimaging and robotic technologies (2). These advances have enabled the design of less invasive techniques, which are more focused, such as deep brain stimulation (DBS) (3). DBS involves electrode insertion into specific neural targets implicated in pathological behavior, which are then repeatedly stimulated at adjustable frequencies. DBS has been used for Parkinson’s disease and movement disorders since the 1960s (4–6) and over the last decade has been applied to treatment-refractory psychiatric disorders, with some evidence of benefit in obsessive–compulsive disorder (OCD), major depressive disorder, and addictions (7). Recent consensus guidelines on best practice in psychiatric neurosurgery (8) stress, however, that DBS for psychiatric disorders remains at an experimental and exploratory stage. The ethics of DBS—in particular for psychiatric conditions—is debated (1, 8–10). Much of this discourse surrounds the philosophical implications of competence, authenticity, personality, or identity change following neurosurgical interventions, but there is a paucity of applied guidance on neuroethical best practice in psychiatric DBS, and health-care professionals have expressed that they require more (11). This paper aims to redress this balance by providing a practical, applied neuroethical gold standard framework to guide research ethics committees, researchers, and institutional sponsors. We will describe this as applied to our protocol for a particular research trial of DBS in severe and enduring anorexia nervosa (SE-AN) (https://clinicaltrials.gov/ct2/show/NCT01924598, unique identifier NCT01924598), but believe it may have wider application to DBS in other psychiatric disorders.

## Background

Anorexia nervosa (AN) has the highest mortality rate of any psychiatric disorder and remains one of the most challenging psychiatric disorders to treat (12, 13). The illness has peak onset in adolescence and is associated with dramatically elevated suicide rates (14), long- and short-term incapacity, and impaired of quality of life (15–17), leading to huge morbidity costs to health services and individuals (18). There is a grave paucity of effective, evidence-based treatments (19–21) and no pharmacological treatments of clear benefit (20–22). Accumulating evidence of conceptual, behavioral, and neural parallels between AN and other “compulsive” psychopathologies (such as obsessive compulsive disorder and addictions) raises the question of whether the compulsive features characteristic of AN (23–26) might respond to deep brain stimulation (DBS) (25, 27–29), in a similar manner to the improvements noted in DBS for OCD (30). Given the shortcomings of current treatments, an increasing number of centers internationally are interested in developing DBS for AN, but it remains an experimental treatment (31, 32) as there are only a few published case reports or series and no controlled studies (33–38).

The application of DBS to individuals with severe and enduring anorexia nervosa (SE-AN) raises particular ethical challenges (39–43), many of which are exacerbated by an invasive experimental intervention on the brain. In particular, issues of autonomy and capacity to consent arise because of the ego syntonicity of symptoms—whereby sufferers often experience the disorder as part of their identity—alongside their apparently paradoxical refusal to engage in life-saving activities (proper nutrition) while claiming a desire to live (1, 9, 44–46). As a result, “compulsory treatment”—which can be controversial—is sometimes employed in clinical practice in order to reduce harm from AN or to save life. Recent legal cases in the UK, and the successful use of guardianship orders in Australia, indicate that judges consider that patients with severe and enduring AN can lack mental capacity to make treatment decisions about their eating disorder, although they are usually found at the same time to possess capacity to make other unrelated decisions. This raises serious questions about the mental capacity of these patients to consent to experimental treatment research into their eating disorder (47–51).

Furthermore, DBS is an invasive brain procedure, and applied to SE-AN it is particularly medically high risk and experimental, with a lack of consensus about optimal neural targets, an increased risk of surgical complications due to severe chronic malnutrition, and a risk of non-compliance due to patients’ ambivalence about recovery (39–43). The risk–benefit calculation fundamental to medical ethics is difficult to make, due to the lack of an evidence base: a few published reports in uncontrolled case series suggest benefit in up to half of individuals with SE-AN undergoing DBS (33–38), but there is also evidence of harm (34)—albeit arguably insufficient to violate the principle of clinical equipoise. However, if the criteria for mental capacity and fully informed consent can be met, and in light of the lack of effective treatments for SE-AN, it can be considered ethically justified to pursue further research into DBS as a potential treatment for SE-AN (18).

In the face of this complexity, we describe a framework of considerations to counter the ethical challenges facing researchers who conduct DBS in SE-AN (Table 1). While our focus is DBS in SE-AN (52), this framework also has implications for other neuropsychiatric conditions and so is likely to have some wider application.

**Table 1:** **The Oxford Neuroethics Gold Standard Framework for deep brain stimulation (DBS) in anorexia nervosa (AN)**.

Assess individual need and possible risk and benefit Given the speculative nature of DBS for severe and enduring anorexia nervosa (SE-AN), will the researchers fully assess the duration of disorder and what previous treatment potential participants have had, and whether there are any conventional treatment options as yet untried? What is the physical and mental status (including comorbid medical and psychiatric conditions) of the potential participants and the level of risk involved in participation in the research? How will the researchers assess these risks? What exclusion criteria will the researchers use (and what criteria for preoperative physical status will they use) to minimize risk while not excluding all potential participants? Consider issues of mental capacity and informed consent Is it acknowledged that mental capacity can be impaired in SE-AN patients and will potential participants be properly assessed for capacity to consent to the research? The researchers must acknowledge this is a particular issue and outline their approach to the issue, as only participants with full capacity should be taking part in this type of novel research. Who is assessing the capacity to consent to research? Ideally this should be an independent ethicist with clinical training in the disorder of interest, or an appropriately trained independent clinician and an independent ethicist working together. It may not be appropriate for the research trial staff to be assessing capacity. What measures or criteria are the assessors using to assess mental capacity to take part in research, bearing in mind mental incapacity can be subtle in this patient group? It should also be borne in mind that some individuals with SE-AN are thought to have capacity in all areas except those relating to the treatment of their disorder. Consider whether impairments of capacity specific to the disorder are present—for AN researchers should assess: Insight and appreciation of the disorder and its impact on oneself; Identity as relating to AN and how this affects decisions regarding treatment and prospect of recovery, for example, what would their identity be without AN; Value systems consistent to AN and how they affect decisions in question; Whether the person feels able to choose to recover or to decide against the impulses of the AN; Whether the person can act against the impulses or compulsions of AN; Ambivalence toward treatment or recovery and the ability to form and maintain a settled decision. How would the researchers ensure fully informed and fully voluntary consent? The researchers should acknowledge the likelihood that potential participants, their families, and their clinicians may feel desperate to try what is usually a last-line treatment strategy. Consider methods of participant support and advocacy during research participation What independent support will the researchers provide the participants to support and advocate for them in situations of distress, anxiety, or difficulties? Will the researchers assess the living arrangements and consider postoperative safety and support of participants? Will the families and partners of participants be involved in supporting participants, and how much will they be informed about the nature and consequences of the trial? Will the research team have an identified contact, such as a surgical research nurse, who can be easily contacted to provide practical advice? Are the surgical and anesthetic teams fully conversant with the needs of SE-AN patients and will the surgical wards be able to support them appropriately during admission, for example, with their eating and dietary needs, and managing compulsive exercise? What are the arrangements for surgical and anesthetic teams, who are usually not used to working with mental health teams, to cowork with the research psychiatrists and participants’ usual mental health professionals? What liaison arrangements are in place for the research team to work with the participants’ usual treating mental health clinicians, especially if the participants are traveling long distances for the research? Consider future care after research participation What are the arrangements for neurosurgical DBS support into the future after the end of the research project, if the participants elect to continue having their DBS equipment *in situ*? What are the arrangements for handing back psychiatric care after end of research? Ethical reflection and support What mechanism will the research team use to reflect on their ethical difficulties? How will the research team learn from their experiences? Consider future public interest in equity of access to the products of any innovation arising from the research

In this paper, we detail the paradigm and gold standard framework, and go on to outline the practical application of the framework, suggesting how it should be used to guide clinical research practice.

## The Oxford Neuroethics Paradigm and Guiding Framework for DBS

### The Oxford Neuroethics Research Paradigm for DBS

**Figure 1:**
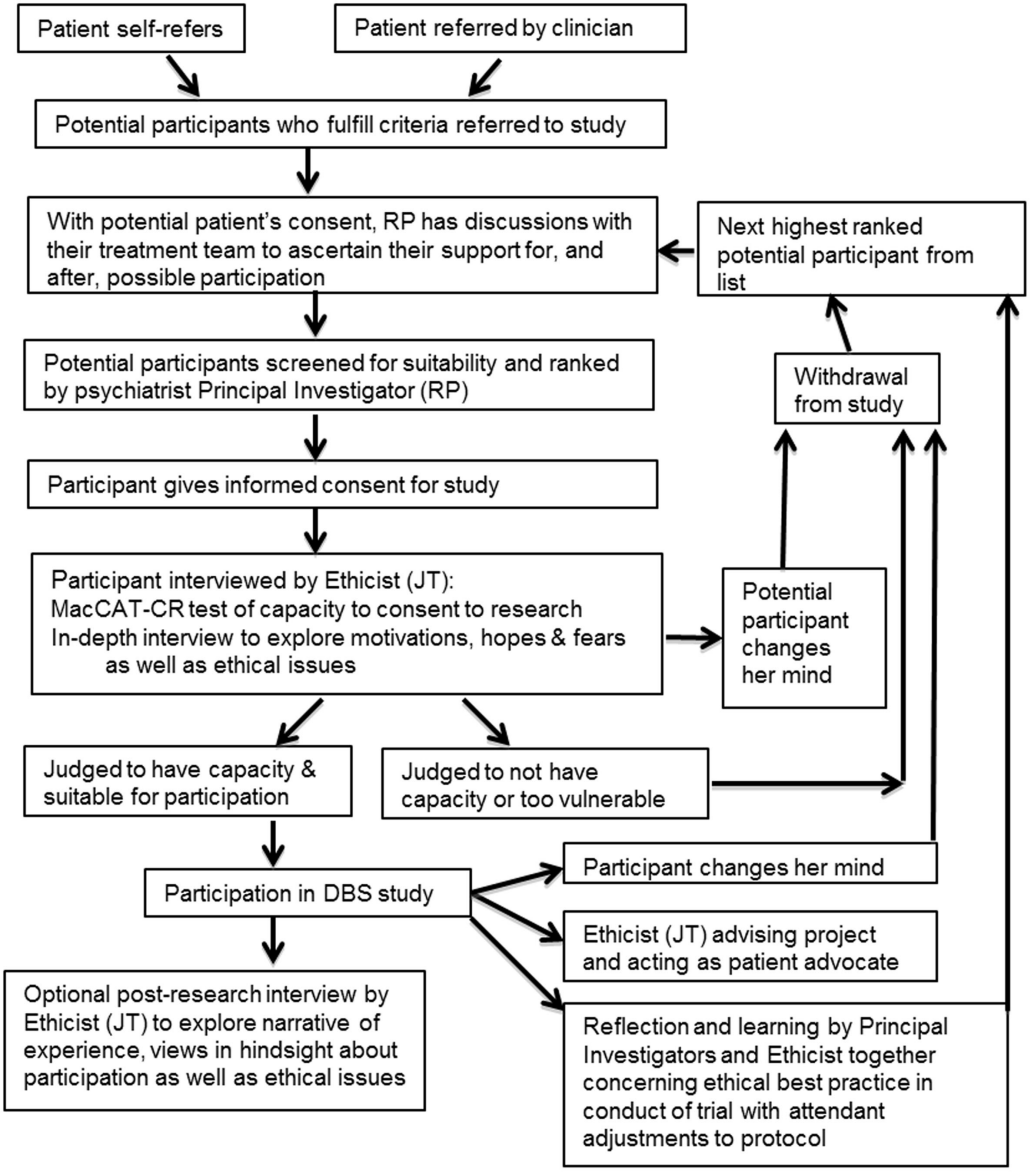
**Diagram of deep brain stimulation (DBS) clinical trial for severe and enduring anorexia nervosa, focusing on the ethical components of this study**. This includes both the ethics sub-study and the checks and balances in the overall protocol.

The Oxford Neuroethics research paradigm was created to support and complement the first UK-registered pilot study of DBS for AN (https://clinicaltrials.gov/ct2/show/NCT01924598, unique identifier NCT01924598), cleared by the Oxford A REC (13/SC/0267). It consists of an ethical sub-study alongside checks and balances at all stages, supported by an independent ethicist who is also an eating disorder psychiatrist. Both the paradigm (Figure 1) and the framework derived from it (detailed in Table 1) are guided by the foundational principles of the Nuffield Council of Bioethics report on “Intervening in the Brain” (53) (detailed in Table 2) (54).

**Table 2:** **An abridged version of The Nuffield Council of Bioethics Ethical Framework for neurotechnology, which we used to inform our Framework (54)**.

**Foundational principles**: Tension between need and uncertainty; Need: suffering caused by disorders and absence of effective treatments; Uncertainty: novelty of neurotechnology and lack of knowledge of mechanisms; The special status of the brain as the organ that gives rise to a sense of self and identity: caution against uncertain effects on personality or sense of self; The special status of the brain as the organ that gives rise to a sense of self and identity: beneficence: The importance of autonomy and dignity-promoting interventions in brain-based disorders.
**6 interests that warrant particular attention**: Protection against the potential safety risks of interventions; Unintended impacts on privacy; Promotion of autonomy; Public interest in equity of access to the products of innovation; Prevention of stigma; Protecting and promoting public understanding and trust in novel neurotechnologies.
**3 virtues guiding research in novel neurotechnologies**: *Inventiveness*, which may be exercised through technological innovation and by identifying ways to provide wider access to therapies (amongst other things); *Humility*, which entails acknowledging the limits of current knowledge and of our capacities to use technologies to alleviate the harms of psychiatric disorders; and *Responsibility*, e.g., pursuit of robust research practices and refraining from exaggerated or premature claims for these technologies.

The paradigm was developed for five distinct reasons:

1. to acknowledge and position ethical considerations centrally within research;
2. to increase the protection of and advocacy for participants;
3. to provide independent guidance to researchers on ethical issues;
4. to better understand the ethical issues (based on the realities of conducting such research);
5. to inform and advance ethical discourse and practice surrounding neuromodulation in severe enduring psychiatric disorders (in particular, SE-AN).

Ethical safeguards were integrated into the protocol from the outset and an independent ethical consultant subcontracted to the project from a different university. Additionally, an ethics sub-study exploring the ramifications of DBS for SE-AN was embedded within the main study protocol from inception. Such a sub-study is recommended by recent consensus guidelines on neurosurgical approaches to psychiatric disorders (8); however, it is not present (to our knowledge) in any other psychiatric DBS research trials worldwide. The inclusion of an independent clinician–ethicist allows for mental capacity and consent to be considered both from a clinical and a philosophical point of view, and the independence of the clinician–ethicist allows decisions to be made that are not at risk of being influenced by the interests of the research over the needs of the patient. The ethics component of the main study and the ethics research sub-study is depicted in schematic form in Figure 1 and can also be found by going to the registered trial online: https://clinicaltrials.gov/show/NCT01924598.

### The Oxford Neuroethics Gold Standard Framework for DBS in AN

Derived from this research paradigm, we recommend the framework outlined in Table 1 to Institutional Review Boards or Research Ethics Committees considering proposals of DBS research in SE-AN. We expect that often, a clinically qualified ethicist with appropriate expertise in the disorder is not available (see point 2b within Table 1 on the assessment of capacity). In these cases, we would recommend that an independent ethicist should work together with an independent clinician, particularly in the assessment of capacity, to ensure that both clinical and ethical inputs are maintained.

## Application of the Oxford Neuroethics Framework for DBS

### Assess Individual Need and Possible Risk and Benefit

Deep brain stimulation research into severe and enduring psychiatric disorders evokes an inevitable tension between the desperation experienced by individuals with chronic and debilitating illness and uncertainty about the safety and the efficacy of DBS in relieving symptoms. This tension can also be considered in terms of the risks and the benefits of DBS for each individual.

#### Medical Risk to Patients

Those with SE-AN are a risky group to perform surgical procedures on, as they have (by definition) chronic malnutrition, very poor physical and mental health, and low quality of life and often require recurrent hospitalizations to stabilize them medically. Both the chronic malnourishment and subsequent micronutrient deficiencies present a challenge to anesthetic and surgical intervention and postoperative wound healing. Neurosurgery can result in epileptic seizures, strokes, and even in rare cases death. In the reported trials of DBS in SE-AN, seizures have been reported in some of the participants (43).

#### Longer Term Risk of Harms

It is also worth considering other less surgical “side effects” that may result, which can range from neurological effects, to mood changes, to personality changes. The concept of “personality” in the face of longstanding chronic illness is complex, as such illness may cloud or color personality function. Notably, some changes in personality function may be seen as a goal of treatment, not a negative outcome of it (8)—but other changes may have a negative effects on relationships and quality of life due to difficulty in transitioning from the role of a patient, adjusting to better psychosocial function with its attendant changes, and a grief for valued aspects or functional purposes (for example, as a safety or coping mechanism) of the illness (10, 55–57).

#### The Benefit of Recovery from AN

The benefits of recovering to any extent from AN are maximal: few treatments are considered efficacious in SE-AN (20, 21), while the costs of illness to the individual and their surrounding system are great. The potential for DBS to ameliorate any of this suffering is perceived as a huge benefit within both the patient population and the clinical community, though notably they may overestimate this potential benefit. This issue will be discussed further in Section “Informed Consent and Desperation in a Clinical Trial.”

#### Etiological Uncertainties

There is a high level of uncertainty concerning the appropriate target for DBS in SE-AN, as the neural basis of the psychopathology remains poorly understood (18). Many neural circuits have been implicated in or speculated upon as contributing to eating disorders (58)—notably those underpinning compulsivity (25) and mood dysregulation (34, 43). Abnormal reward processing and compulsivity in AN implicate striatal targets such as the nucleus accumbens (59)—a DBS target for addictions and OCD (60), while mood dysregulation and dysphoria suggest DBS targets used in depression, such as the subcallosal cingulate (18, 34, 61). At this stage, it remains unclear which—if any—DBS neural target will provide optimal results with minimal risks in AN, or indeed, whether different areas will provide different benefits for different patients.

#### Outcome Uncertainties

As psychiatric disorders are based on complex neural circuitry and interactions with psychosocial factors, any symptomatic change is unlikely to be as dramatic as documented with movement disorders. In contrast to the sudden improvements in symptoms in Parkinson’s, in those AN patients who respond to DBS of the subcallosal cingulate (34), improvement is gradual and indirect: improved mood regulation appears to enable reduction in ED symptoms such as binge purging, and subsequent reengagement in treatment programs that have previously been unsuccessful (34). It is less clear what the time course of response to compulsivity/reward processing targets such as the nucleus accumbens would be (18); one clue is that in treatment-resistant OCD, DBS to the nucleus accumbens, similarly to DBS of the subcallosal singulate, appears to allow CBT to become effective (30).

It is of key importance to consider with patients the renewal of desperation or the disappointment, which may result from no or minimal improvement in AN symptoms after DBS (9, 44, 62–64), and to stress that DBS should not be considered as a stand-alone “miracle cure,” but rather an intervention that might facilitate response to other treatment modalities such as psychotherapies and weight restoration programs—which may not have been previously tolerated or effective. Discussion in the section “Informed Consent and Desperation in a Clinical Trial” of the therapeutic misconception draws strongly on the discussion of the uncertainties in this section and the previous one.

#### Necessary Safeguards

Given these complex potential risks and benefits, in order to reduce the surgical and psychological risks to potential participants it is thus crucial to assess suitability by exploring each individual’s deeper understanding, motivation, desires, and reality testing, as well as prior and recent personality functioning, and physical and mental state.

Strong safeguards in the protocol regarding potential safety risks are essential, including continuous critical examination of unanticipated or unknown safety risks. All potential participants should be carefully screened for their suitability. Given operative risks in medically unstable and severely malnourished individuals, a body mass index over 13 with physical parameters held relatively stable is recommended. Careful monitoring of participants’ physical and psychological health should take place throughout the study, with psychiatric-neurosurgical follow-up offered beyond the protocol period if the individual opts to keep DBS *in situ*. Participants should receive care from the neuropsychiatric/neurosurgical team in addition and in collaboration with their normal clinical treatment team.

Safety considerations necessitate platforms that enable quick, transparent, and accurate communication, as well as transfer of relevant data between these teams. Carers and family must be supported, recognizing that unpredictable risks of the DBS procedure are endemic to the SE-AN population, due to the consequences of severe starvation and AN-related behaviors (such as binging and purging), and also from consequences of comorbid psychiatric disorders (65).

### Consider Issues of Mental Capacity and Informed Consent

#### Competence to Consent to Research in AN

It is important to consider that those with SE-AN may have impaired mental capacity, and especially that while they may have an intact intellect, it might be considered that they are not capable of making decisions about their disorder, or be competent to (66). This has also been recognized in legal cases in recent years (49–51). Therefore, competence to give consent to research needs to be properly and independently assessed, using suitable tools. Research suggests that competence can be impaired in several ways: people who have AN may feel that the disorder is part of their identity; they may adopt the values of AN so that, for example, being thin is more important than life itself; they may find themselves unable to make a choice to recover from the disorder even if they wish to; and they may find themselves unable to act upon the choices that they make (66–69). A structured approach of in-depth assessment of capacity in an eating disorder is provided in Table 3. We recommend using a standard tool such as the MacCAT-CR, followed by an in-depth exploration of motivations, hopes, and expectations (70).

**Table 3:** **Format for the assessment of capacity in eating disorders, including how one might assess capacity within the context of an intervention**.

1. Assess ability to understand and retain information	Checking understanding and retention is fairly straightforward—disclosure can be followed by a request for the patient to repeat the information back in his or her own words. The MacCAT-T clinical competence instrument provides a structured and systematic framework for doing this (71). There is a version specifically for competence to consent to research, the MacCAT-CR (70).

2. Assess ability to use information	This can be assessed in the course of the discussion and by asking the patient for his/her reasons for the decision—it should become evident whether the patient is able to use the information provided.

3. Assess appreciation of information and facts of the decision	Appreciation, not seen in UK legislation but found in Grisso and Appelbaum’s definition of competence, is the ability to apply the information to oneself (71). This can be a problem in eating disorders, for example, a patient may say, “I understand that’s the definition of an eating disorder, I understand I have those features, and I understand eating disorders need treatment; but I do NOT have an eating disorder and therefore I do not need treatment.” This clearly would affect capacity to make decisions about treatment for an eating disorder.

4. Assess presence of compulsion	Look for compulsions (or obsessions) that may prevent the patient from acting on the basis of his/her understanding or even desires. The Code of Practice of the Mental Capacity Act gives an example that patients with anorexia nervosa (AN) may be unable to “use and weigh” treatment information as part of the decision-making process: “For example, a person with the eating disorder AN may understand information about the consequences of not eating. But their compulsion not to eat might be too strong for them to ignore” (72).

5. Assess for changes in values due to the eating disorder	It is part of the core criteria of AN that a person should either have a fear of fatness, or an overvaluing or pursuit of thinness (73). This dread of fatness and overvaluing of thinness, found in many eating disorders, means that being thin or losing weight becomes disproportionately highly valued by sufferers, in some cases this is even valued above life itself. This disproportionate value can drive some patients to decide, even after they have weighed up the options, not to have treatment because they would rather die than gain weight (66).

6. Assess for changes in identity due to the disorder	One of the characteristics of eating disorders is that they can be ego-syntonic, that is, the disorder is experienced as part of the self and also consistent with one’s own values (57). Further, many people with eating disorders become ill as adolescents and may as adults have little or no sense of who they would be without the disorder. This intertwining of the disorder with the sense of self can make it difficult to decide to have treatment in order to recover from it; for example, patients may be either unable to envisage a self without eating disorders (66).

7. Assess for depressive features, loss of hope, and affective elements	Eating disorders have clear effects on emotion and mood; there is a high rate of comorbidity of depression (74). It is important to assess for depressive features and particularly for suicidality, more covert wishes for death (for example, wanting to die thin) and inability to envisage or hope for recovery, all of which would affect how options are weighed. Charland and colleagues further argue that beyond comorbid depressive disorder, AN itself may have clear affective components, fitting Ribot’s conception of a “passion” in its very nature (75). These components include having a fixed focus and motivational force and attachment; these may have an impact on decision-making.

*This touches on issues such as compulsion, depression, and changes in values due to the eating disorder*.

#### Informed Consent and Desperation in a Clinical Trial

It is important that full and graphic information and education about the purpose of the clinical trial is given to each patient, leading to fully informed, voluntary consent. Consent, even if it is without coercion and fully informed, may be constrained in terms of lack of alternative choices (63). In this context of “last-line” treatment research, with the attendant desperation and lack of choice, it may prove impossible to avoid all misinterpretations, leading to inadequate consent (45). Desperate patients may also lack the critical faculties to properly interpret what they read in the media about DBS, or misinterpret the nature of the trial (45). It is thus essential to stress that in such experimental treatment research, there can be no guarantee that a patient will benefit. Rather, it is expected that more general benefits will accrue, such as an improvement in knowledge, or future treatment options. Fully informed consent must involve the recognition of these nuances and the discussion of how DBS should not be regarded as a “miracle cure.” Researchers must engage in complex discussions with prospective participants about the current situation regarding evidence for DBS, particularly the etiological and outcome uncertainties outlined previously. This is because a failure to appreciate this would almost certainly lead to difficulty in appreciating the underlying principle of equipoise, which governs the ethical approval of clinical research employing DBS. Without this complex understanding, it would be difficult to safely judge that potential participants do indeed possess the capacity to consent to the research. The main risk is “Therapeutic Misconception,” a misapprehension that the research clinicians are performing the procedure with the expectation of benefit to the individual (76, 77).

Even with a full understanding of the primary purpose of research as not for personal benefit, the uncertainties involved, and the risk of harm without benefit, patients who feel they have no other options available to them may be highly motivated to commit to high risk for even low chances of recovery, leaving them vulnerable to exploitation. The extent to which patients perceive benefit from this experimental treatment is further illustrated in patients’ pursuit of these novel treatments. Anecdotal evidence from DBS researchers suggests that families and patients often proactively ask for the opportunity to take part in experimental research. Some patients show commitment to involvement to the extent that they gain weight in order to participate in research, even when previously weight gain appeared impossible. Others have offered to relocate or travel regularly across the country or internationally in order to be able to take part in DBS trials (personal communications and our experience).

### Consider Methods of Participant Support and Advocacy during Research Participation

#### Patient Advocacy and Support

The integration of an independent ethicist from an external institution allows the participants to have external support, and also these ethicists may act as advocates for carers and families. The provision of an independent clinician and/or ethicist also ensures that patient feedback can be integrated into decision-making and provides both researchers and participants with opportunities to obtain support, advice, and a third-party opinion.

#### Returning Home: Living Environment

After DBS treatment, patients will have to return home and continue with their normal lives. The living environment and recovery of participants must be considered; they should be placed in an environment that is conducive to healing, is supportive, and where help is available. The environment should, if possible, not be one that is triggering to their eating disorder, and ability to contact other family members and clinical teams must be considered. Additionally, proximity to hospitals or medical care should also be discussed, especially in the early stages of healing.

#### Family Involvement

It is also necessary to consider to what extent families will act as carers or supports for the participant and to discuss with the participant how they will be involved and what they should be informed of. At a minimum, they should be informed of risks and symptoms to watch out for during the recovery period, such as rapid mood changes (mania or depression) or signs of infection or poor healing. Furthermore, medical aftercare must be considered before the procedure begins—a clear path for participants or their families to follow if participants experience any negative effects or have any medical concerns must be developed. It should also be borne in mind that it is highly likely that families, along with patients, are likely to be desperate for improvement and may be as susceptible to the “therapeutic misconception” and suffer from dashed hopes as much as their loved ones.

#### Surgical Teams and AN Patients

Consider also that patients who have DBS must be admitted to neurosurgical wards where staff may be unused to dealing with patients with severe psychiatric disorders. Psychiatric patients tend to get poorer care from, or be stigmatized by, health professionals in general medical and surgical settings (78), who may misunderstand their difficulties with eating, or see their illness as self-induced (79). Psychiatric liaison, support, and training of the neurosurgical teams involved are thus crucial. Once a patient is deemed postoperatively stable by the neurosurgical team, it may be beneficial to admit them to a specialized medical-psychiatric unit, if available, to mitigate this difficulty.

#### Privacy

Protection of privacy is paramount in all patients, but patients participating in research on DBS for SE-AN may be at unusual risk of privacy violations for a number of reasons. The overall number of patients undergoing this treatment in any country is very small, and the clinics pursuing research on DBS in SE-AN will be easily identifiable. Anorexic DBS patients are also visible to the public in a prosaic way as a consequence of their severe AN, in combination with the visibility of DBS apparatus *in situ*. Moreover, there is a high level of media interest in DBS in AN. The UK media has already exploited the “news value” of DBS in SE-AN with a significant degree of bias (80). Thus, patients’ identities are easily revealed either deliberately through media investigation, or inadvertently through their social circles. Participants who have identified themselves (34, 81) may subsequently find continued public scrutiny intrusive or burdensome, or individuals may be identified without their consent and subjected to scrutiny that they are not prepared to cope with. The high potential for privacy violations for this patient population requires research teams to take great care when publicizing their research, particularly in the media, but also more generally on university web platforms and in the community. The potential for these privacy violations to lead to stigmatizing the individual is also high, and participants must be informed of the possibility of this and be supported through any such occurrences.

### Consider Future Care after Research Participation

There are long-term care considerations and cost implications in experimental treatment research into DBS, due to requirements for lifelong neurosurgical follow-up and device maintenance. It would be ethically dubious, given the high costs of AN, to submit an individual to DBS insertion and its risks only to remove it after the end of a study protocol period if the individual had experienced benefit. It is thus essential that long-term, post-protocol arrangements are specified. For patients with SE-AN who may only be in their 20s or 30s at the time of surgery, there is potentially a requirement for half a century or more of follow-up and maintenance care. This demands a health-care system that is able to absorb the cost of DBS follow-up as part of the “routine medical care” of the patient; an issue that is likely to be problematic in countries where health-care is either privately funded, insurance-based, or over-stretched, or where patients may return or migrate to countries that cannot support appropriate aftercare. These questions should be considered before any trial is begun, and the appropriate agreements should be sought.

Once participants have returned home, it is necessary that their psychiatric care continues. Even if they receive benefit from DBS, it is highly possible that there will be adjustment problems, as has been seen in Parkinson’s disorder. This is known as the “burden of normality,” and results from the “best case scenario”—a participant receives benefit from DBS (82). Psychiatric support is needed up until the point where the participant can resume normal life and to ensure that improvement persists and that adjustment is smooth. If no benefit has accrued, then there may be deep devastation and loss of hope, especially if participants saw DBS as a “last-line” treatment. Therefore, long-term psychiatric care arrangements must not be overlooked and are of crucial importance.

### Ethical Reflection and Support

While DBS for AN is an exciting and novel treatment development, we have noted above a number of ethical difficulties, which may be encountered. The enthusiasm and potential naivety of researchers to this type of research should thus be tempered. This is best managed by creatively incorporating ethics into the heart of DBS research, as opposed to planning research that is sufficiently ethical to meet standards set by research ethics committees.

Along with the advocacy and participant liaison benefits of contracting an independent researcher(s) to the study, this demands that the researchers explain and justify any decisions to the external ethicist/clinician. This need to explain decisions allows a point of reflection and means the researchers have to be accountable on an everyday basis, in addition to their general accountability to the research governance processes. By integrating the ethicist in discussions about decisions, the researchers open themselves to the vulnerability of contemplating the responsibilities they bear and the ramification of these responsibilities.

Reflection can also be achieved by following the example of the Oxford Neuroethics Research paradigm, which incorporates an ethics sub-study component into the protocol. This allows researchers, through exploring the ethical issues with the participants and capturing their experiences and narratives, to contribute empirical data to advance the field of research ethics within the area of DBS treatment.

### Consider Future Public Interest in Equity of Access to the Products of Any Innovation Arising from the Research

Even if the research is at this preliminary stage, it is important to consider future considerations of equity if there are products of the research innovation.

The principle of justice in relation to DBS research is little discussed (63). However, equity of access is a major problem in DBS for SE-AN. As a highly speculative, expensive, and specialist treatment, it is currently only available within research trials, based in a limited number of neurosurgical specialist centers. These trials are highly limited in numbers due to the difficulty in funding such research. Arguably, this may be no different to the issue of potential participation in a trial of any new intervention, which is always dependent on the serendipity of being a patient of a recruitment center, or living in the recruitment area. However, this inequity is likely to continue even if DBS for SE-AN acquires the evidence base to become a standard treatment, because it is unlikely that countries or regions that lack the requisite high levels of health-care funding or specialist neurosurgical expertise would be able to offer it routinely. It has been recommended that country of origin, along with many other factors, should not affect whether a novel neurosurgical treatment is offered (8)—but implementing this in practice is challenging.

The potential for participants to pay for their own DBS treatment within the trials or in the future also raises questions of equity. Such self-funding may be less unacceptable or exceptional in the context of a privately funded health-care system, where patients and the public accept the concept of accessing treatments according to personal wealth. However, this may be deemed more unacceptable in a health-care system where other treatments are publicly funded and free at the point of delivery—and therefore considered an individual’s right—such as in the UK.

## Conclusion

We have presented an innovative neuroethical framework applied to DBS research in SE-AN to guide both research ethics committees and researchers in neurotechnologies applied to vulnerable psychiatric populations. Critically, this framework should always be applied to research in SE-AN, due to the special concerns it merits in relation to physical health and capacity. It incorporates ethical gold standards in synergy with a means of empirically investigating the ethics of such research. We suggest that this method of incorporating ethics into the heart of novel neurotechnology research ensures that rigorous day-to-day ethical input is available within such sensitive and difficult research, and the welfare of such vulnerable participants is maximally protected. We also believe that it exemplifies the Nuffield Council of Bioethics’ recommendations regarding the ethics of novel neurotechnology (2013) (53). The practical ethical issues that arise in this sort of research are as yet not fully investigated, and it is our hope that this research can not only extend the boundaries of DBS science but also contribute to the field of ethics of research.

## Ethics Statement

The clinical trial discussed in this paper is being carried out in accordance with the recommendations of the Oxford A Research Ethics Committee, with written informed consent from all subjects. All subjects gave written informed consent in accordance with the Declaration of Helsinki. The protocol was approved by the Oxford A Research Ethics Committee.

## Author Contributions

Creation of the paradigm for the DBS clinical trial discussed in this paper, including the ethical sub-study: RP and JT. Conception and design of Oxford Neuroethics Gold Standard Framework; drafting the article and revising it critically for important intellectual content: RP, IS, AP, and JT.

## Conflict of Interest Statement

The authors declare that the research was conducted in the absence of any commercial or financial relationships that could be construed as a potential conflict of interest.
